# Bull Trout (*Salvelinus confluentus*) Population Structure Across Alberta's Eastern Slopes

**DOI:** 10.1002/ece3.72761

**Published:** 2026-02-24

**Authors:** Emily R. Franks, Benjamin C. Kissinger, Steve Amish, John R. Post, Jonathan A. Mee

**Affiliations:** ^1^ The University of Calgary Calgary Alberta Canada; ^2^ fRI Research Hinton Alberta Canada; ^3^ Montana Conservation Genomics Lab Missoula Montana USA; ^4^ Mount Royal University Calgary Alberta Canada

**Keywords:** bull trout, hierarchical population structure, landscape genetics, *Salvelinus confluentus*

## Abstract

Understanding a species' genetic population structure is fundamental for determining its conservation and management units. Bull trout ( *Salvelinus confluentus* ), a charr native to northwestern North America, have experienced significant population declines across their range. In Alberta, bull trout are classified as Threatened under Alberta's *Wildlife Act*, but knowledge of their genetic population structure is limited. We aimed to assess bull trout population structure in Alberta using genetic markers from a RADcapture SNP panel. Our samples spanned 24 Hydrologic Unit Code 8 (HUC 8) watersheds across Alberta's Eastern Slopes region. One of our goals was to evaluate support for two previously proposed bull trout Designatable Units (DUs) in Alberta: the Western Arctic DU and the Saskatchewan‐Nelson DU. We found notable population structure and high differentiation (*F*_ST_ = 0.400) between these two DUs, suggesting two discreet populations that correspond to the northern and southern biogeographical zones of Alberta. We also conducted *ad hoc* population differentiation analyses using ADMIXTURE, which revealed that bull trout have a hierarchical (or nested) population structure. Our results inform management of the species and suggest protecting the local adaptations and genetic diversity of bull trout at both broad and fine‐scale spatial scales.

## Graphical abstract

To understand the genetic population structure of native bull trout (*Salvelinus confluentus*) for conservation and management actions, we performed a genetic analysis on 962 bull trout samples collected across Alberta's Eastern Slopes. Our results found evidence for two distinctive populations, which correspond to the two bull trout Designatable Units: the Western Arctic DU and the Saskatchewan‐Nelson DU. We also found bull trout express a “hierarchical metapopulation structure” at a finer scale, which provides insight into the conservation and management of the species.

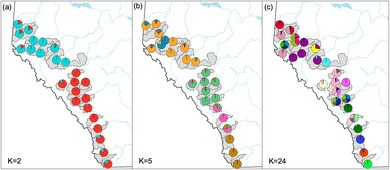

## 1 Introduction

Aquatic species across the world are facing declines in distribution and abundance under stress from warming waters, pollution, over‐harvest, and habitat degradation, which all threaten global biodiversity (UNEP 2021; IPCC 2022; WEF 2024). Managing freshwater species, however, can present significant challenges because the risks associated with a species' decline may differ widely among various populations or regions within a single species. In Canada, wildlife are assessed in conservation units called Designatable Units (DUs), which are defined as populations having unique adaptations or discrete evolutionary significance (COSEWIC 2020). Such units are considered irreplaceable and, therefore, deserving of independent protection (COSEWIC 2020).

The identification of conservation units, such as DUs, requires understanding a species' genetic population structure as it's essential to guide the identification and management of locally adapted or evolutionarily significant populations (COSEWIC 2020). Understanding spatial genetic population structure is also crucial for assessing connectivity and abundance, which contributes to a species' population status (Hudson et al. 2017). This ultimately contributes to identifying and mitigating undesirable effects, such as from intraspecific hybridization (Harbicht et al. 2014; Roques et al. 2018), and for appropriately planning conservation actions such as conservation translocations (Minckley 1995; Galloway et al. 2016) and restoration stocking (González‐Wangüemert et al. 2012). For example, choosing appropriate strains for restoration stocking or conservation translocations requires understanding the species' genetic structure to ensure the strains being introduced are similar to the wild populations being supplemented or existing in connected waters, which is essential for protecting local adaptations and genetic diversity (Galloway et al. 2016).

Bull trout ( *Salvelinus confluentus* ), a freshwater charr species endemic to northwestern North America, have faced population declines across their native range in both Canada (Government of Canada 2024) and the USA (USFWS 2015). Bull trout require cold, clear, and connected water and typically spawn in early fall. Since bull trout have a narrow range of habitat requirements, increased warming of waters is likely a significant impact for the survival and persistence of bull trout (Eby et al. 2014). The most significant threats to bull trout populations currently include habitat degradation and fragmentation of rivers or streams, climate change, and the introduction of non‐native species (Reiman et al. 2007; Vander Zanden and Olden 2008; Young et al. 2016; Sinnatamby et al. 2020; Bell et al. 2021).

Bull trout are known to exhibit strong site fidelity, where fish migrate to lakes, main stem rivers, and, in rare cases, marine environments for foraging, but return to their natal sites to spawn (Fraley and Shepard 1989; Howell et al. 2016). This natal homing in bull trout, as in many salmonids, often results in highly spatially structured populations (Potvin et al. 2003; Taylor et al. 2003; Warnock et al. 2010; Hagen et al. 2021). At their spawning sites, female bull trout build redds in small streams or creeks in the fall and, following a brief courtship, lay eggs in the redds immediately prior to fertilization by a male. In the following spring, small alevin begin their lives as either fluvial, adfluvial, or migrant individuals (Stewart et al. 2007). A few sea‐going migratory populations are found in coastal regions, but the majority of the spawning habitat of bull trout is in the high‐elevation headwaters in the interior mountainous regions of Western North America (Taylor et al. 2001; Costello et al. 2003).

At the broadest scale, bull trout are comprised of two main lineages found throughout Western Canada and Northwestern USA (Whiteley et al. 2019). These two lineages likely originated within the Columbia River refugium and the Chehalis River refugium during the last glacial maximum in the Pleistocene (Spruell et al. 2003; Ardren et al. 2011). Geographic isolation in each refugium likely led to the formation of the two genetically divergent lineages: “Genetic Lineage 1” and “Genetic Lineage 2” (Leary et al. 1993; Taylor et al. 1999). After the retreat of the Cordillera Ice Sheet, these populations colonized the deglaciated regions to the north, giving rise to the modern‐day bull trout lineages found in interior mountain ranges today (Taylor et al. 1999).

**FIGURE 1:**
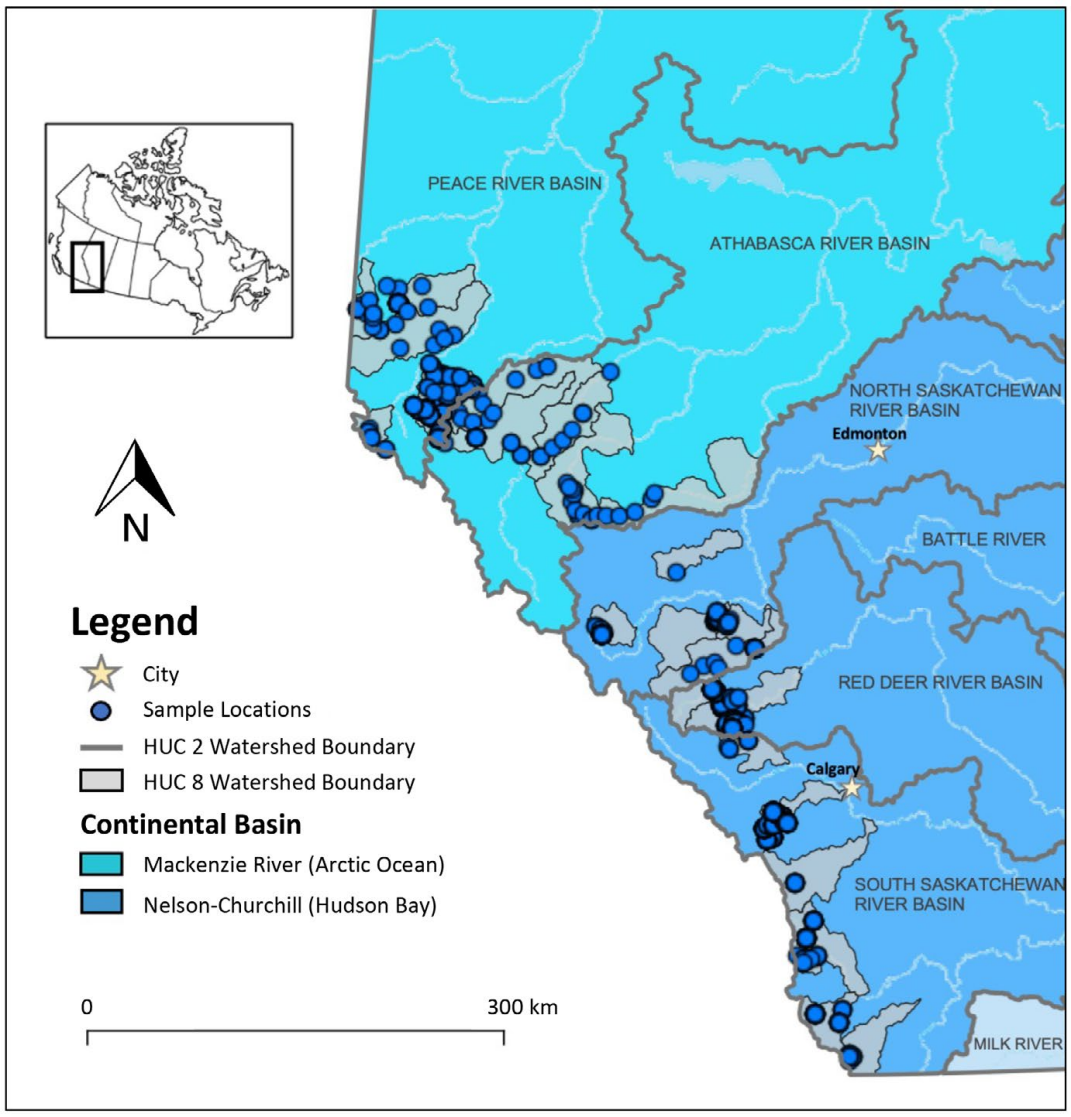
Locations of bull trout (*n* = 962) samples spanning 24 Hydrologic Unit Code (HUC) 8 watersheds across the Alberta Eastern Slopes by Alberta Environment and Protected Areas staff and volunteers between 1999 and 2022.

In Canada, five bull trout DUs have been identified (COSEWIC 2012). The Southern British Columbia DU consists of bull trout from Genetic Lineage 1, whereas Genetic Lineage 2 is comprised of the other four DUs (COSEWIC 2012). Our study focuses on understudied aspects of bull trout population structure in Alberta and current evidence suggests that two bull trout DUs are present in Alberta: the Western Arctic DU and Saskatchewan‐Nelson DU. These two DUs correspond to the two major continental‐scale watershed basins in Alberta, where the former eventually flows to the Arctic Ocean via the Mackenzie River and the latter eventually flows to Hudson Bay via the Nelson River (Figure 1). Presently, the Western Arctic DU is listed as “Special Concern” and the Saskatchewan‐Nelson DU is listed as “Threatened” (COSEWIC 2012).

While the genetic structure of bull trout has been studied extensively in BC and the USA (Leary et al. 1993; Taylor et al. 1999; Latham 2002; Costello et al. 2003; Ardren et al. 2011), bull trout genetic diversity and population structure across the Eastern Slopes of the Rocky Mountains in Alberta, where conservation risk is most pronounced, have not been sufficiently characterized to inform management across the province (Warnock et al. 2010; Carroll and Vamosi 2021). An assessment of bull trout from 20 sites within the Athabasca River and North Saskatchewan River basins in Alberta showed that bull trout across these two drainages are genetically differentiated, validating the designation of two bull trout DUs on either side of the continental‐scale divide between these basins (Carroll and Vamosi 2021). Additionally, these results indicated bull trout population structure is hierarchically structured (Carroll and Vamosi 2021), which is consistent with other findings on the genetic structure of bull trout (Spruell et al. 2003; Warnock et al. 2010). However, many watersheds to the north and south of these areas have not been assessed, compromising our ability to characterize the scale at which to develop conservation policy and management actions for this culturally and ecologically important species. Additionally, an analysis using microsatellites on bull trout in Pine Lake, Peace River drainage BC found these populations had lower genetic diversity relative to bull trout in Southern BC (Costello et al. 2003), suggesting populations on the periphery of the bull trout range, which are more recently colonized than those closer to their pre‐colonization refugium, may generally have lower genetic diversity. Given that the Pine Lake BC population belongs to the same Western Arctic drainage as is found in Alberta, it is possible that bull trout in Alberta also have lower genetic diversity relative to Southern BC and other parts of the bull trout range.

Fine‐scale population sampling, such as within‐watershed, is necessary to shed light on the scale at which evolutionary processes, such as local adaptation, gene flow, or genetic drift, drive population divergence (Warnock et al. 2010). This is essential in the potential management of bull trout, such as supplementing bull trout populations for recovery (Hansen et al. 2009), which would require thoughtful consideration of genetic structure to protect local gene pools from non‐natural genetic drift (Cross 2001) or intraspecific hybridization (Hansen et al. 2009), a consideration that is especially critical in smaller populations that are more vulnerable to impacts on their gene pools (Meffe 1986). Additionally, as fish in interior freshwater systems typically have fewer migratory overlaps and are more genetically isolated (Ward et al. 1994), genetic subdivisions for freshwater fish are likely to be more complex than those for coastal or migratory species, making fine‐scale population assessments essential for the conservation of interior freshwater species.

The goal of this study was to investigate the population structure and genetic diversity of bull trout across various spatial scales in the Eastern Slopes of the Rocky Mountains in Alberta. We used single nucleotide polymorphisms (SNPs) obtained from a RADcapture panel that was designed to capture high‐resolution variation within the bull trout genome (Amish et al. 2022). We assessed various a priori hypotheses for population structure based on the two DUs (Figure 1) and various Hydrologic Unit Code (HUC) scales, which are watershed‐level scales used for management across the province. In Alberta, major river basins are managed at a HUC 2 scale, which includes the Peace River, Athabasca River, North Saskatchewan River, the Red Deer River, and the South Saskatchewan River basins, whereas local or regional management is often focused at a HUC 8 scale or smaller for this species, which are watershed‐level management units nested within the HUC 2 boundaries. We also conducted *ad hoc* analyses to identify population structure that may exist at other scales. Additionally, to test whether genetic divergence between populations is correlated with geographic distance, we evaluated the existence of isolation by distance. Our study is the first range‐wide assessment of population structure and genetic diversity for bull trout across Alberta.

## 2 Materials and Methods

### 2.1 Sample Collection and Genotyping

Samples were collected and archived under fisheries research license's applied for and provided to staff from various agencies by the Government of Alberta. Tissue samples (non‐lethal fin clips) were collected while conducting standard fisheries assessments to estimate population abundance by Alberta Environment and Protected Areas (AEPA) staff between 1998 and 2022 (Table S1) using either gill netting, electrofishing, or angling. These dried fin clips were then archived for future analysis. Data from those sample archives were then opportunistically used in this study. A total of 1281 Salvelinus samples (putative bull trout, brook trout, and their hybrids) were collected from 24 HUC 8 watersheds in the Peace River (Western Arctic DU), Athabasca River (Western Arctic DU), North Saskatchewan River (Saskatchewan‐Nelson DU), Red Deer River (Saskatchewan‐Nelson DU), and South Saskatchewan River basins (Saskatchewan‐Nelson DU; Figure 1). Fin clips were sent to the Montana Conservation Genomics Group for genotyping using a RAD‐seq SNP panel designed for bull trout consisting of high‐quality variable SNP markers (Amish et al. 2022). The SNP panel was developed from samples spanning bull trout's North American distribution and included samples from all 24 HUC 8 s analyzed in this study, thereby reducing any chances of ascertainment bias that may otherwise be present when using samples from only one location or jurisdiction. In a prior study focusing on the extent of hybridization between bull trout and brook trout, we determined that, of the 1281 *Salvelinus* samples, 962 individuals had no brook trout ancestry (Franks et al. 2025). We used only these 962 bull trout in the present study for our analysis of population structure and diversity (Table 1). Using a set of 1039 loci among the 962 bull trout samples in our study, we filtered for Linkage Disequilibrium (LD) by calculating pairwise *r* ^2^ (Pearson correlation coefficient) using the *genetics* package in R (Warnes et al. 2021; Figure S1). Loci were then trimmed using PLINK (Purcell et al. 2007) if they exceeded an *r* ^2^ threshold ≥ 0.5. We investigated other threshold values for LD pruning (i.e., *r* ^2^ threshold ≥ 0.2, 0.4, 0.6, and 0.8), which produced variable results in terms of the number of SNPs retained for subsequent analysis (Figure S1), but this choice did not affect patterns or qualitative interpretation of subsequent results. Monomorphic loci were also removed if they lacked variation across all populations prior to further analyses.

**TABLE 1:** Bull trout sample counts and observed heterozygosity (Ho), expected heterozygosity (He), and Allele Richness (Ar) from 24 Hydrologic Unit Code (HUC) 8 watersheds across Alberta, spanning five HUC 2 river basins in the Western Arctic (WA) and the Saskatchewan‐Nelson (SN) Designatable Units.

	HUC 8	HUC 2	DU	Bull trout samples (*n*)	Ho	He	Ar
1.	Cutbank	Peace River	WA	22	0.0981	0.0964	1.29
2.	Narraway	Peace River	WA	10	0.1149	0.1077	1.23
3.	Kakwa	Peace River	WA	9	0.1069	0.1161	1.23
4.	Muskeg	Peace River	WA	98	0.0349	0.0443	1.32
5.	Sulfur	Peace River	WA	112	0.0543	0.0708	1.30
6.	Jackpine	Peace River	WA	7	0.0735	0.0730	1.15
7.	Berland River	Athabasca River	WA	10	0.0466	0.0482	1.10
8.	Upper Athabasca & Oldman	Athabasca River	WA	10	0.0822	0.0908	1.19
9.	Wildhay	Athabasca River	WA	14	0.0466	0.0444	1.11
10.	Upper McLeod	Athabasca River	WA	10	0.0457	0.0452	1.08
11.	Upper Pembina	Athabasca River	WA	8	0.0169	0.0168	1.03
12.	Nordegg	North Saskatchewan River	SN	1	0.0825	n/a*	n/a*
13.	Ram	North Saskatchewan River	SN	37	0.0858	0.0879	1.16
14.	Prairie	North Saskatchewan River	SN	8	0.0791	0.0651	1.06
15.	Cline	North Saskatchewan River	SN	39	0.0417	0.0478	1.20
16.	Clearwater	North Saskatchewan River	SN	16	0.0881	0.1004	1.28
17.	Panther	Red Deer River	SN	76	0.0830	0.0862	1.31
18.	Upper Red Deer	Red Deer River	SN	181	0.0857	0.0899	1.34
19.	Ghost	South Saskatchewan River	SN	7	0.0464	0.0656	1.06
20.	Elbow	South Saskatchewan River	SN	189	0.0310	0.0393	1.12
21.	Highwood	South Saskatchewan River	SN	7	0.0548	0.0577	1.05
22.	Upper Oldman River	South Saskatchewan River	SN	49	0.0405	0.0396	1.23
23.	Castle	South Saskatchewan River	SN	27	0.0506	0.0548	1.17
24.	Waterton	South Saskatchewan River	SN	15	0.0539	0.0506	1.12
	**Overall**			**962**	**0.0636**	**0.0669**	**1.13**

^*^ Unknown values due to Nordegg having *n* = 1 sample size.

### 2.2 Population Structure and Diversity Analyses

We performed *ad hoc* bull trout population identification based on the LD‐filtered SNP dataset using ADMIXTURE (Alexander et al. 2009), which estimates ancestry proportions for each individual. We set the number of putative populations (*K*) from 1 to 25 and used a cross‐validation error plot to determine the most likely value of *K*. *K* of 25 was selected because it represents one more than the total number of HUC 8 watersheds present within our study area. A principal component analysis (PCA) was also performed on all bull trout (BLTR) samples by first converting SNP data to genotype file data using PLINK (Purcell et al. 2007). PCA scores for each sample were calculated using the *gdsfmt* and *SNPRelate* (Zheng et al. 2012) packages in R (R Development Team 2024). The first two principal component eigenvectors were then used to inspect the greatest variance among individual samples and watersheds.

Within each putative population, we used the R packages *SNPRelate* and *pegas* (Zheng et al. 2012) to identify and remove loci that deviated significantly from Hardy–Weinberg equilibrium (HWE) (Bonferroni‐corrected *p* > 0.05) using the “Out Any” approach (Pearman et al. 2022) before calculating genetic diversity metrics. The observed and expected heterozygosities (Ho & He) and allele richness (Ar) using the mean number of alleles per locus rarefied to 2 times the size of the smallest population were calculated for each putative population using *adegenet (*Jombart 2008) and *hierfstat* (Goudet 2005) packages in R (R Development Team 2024).

Genetic divergence (*F*_ST_) was estimated using the *adegenet* package in R (Jombart 2008). Bull trout genotypes were first converted into a “genind object” and then *F*_ST_ was estimated using the Weir and Cockerham (1984) method (Weir and Cockerham 1984). We calculated pairwise *F*_ST_ at several scales: between the two DUs (Western Arctic and Saskatchewan‐Nelson), among the five HUC 2 watersheds (Peace, Athabasca, N. Sask, Red Deer, and S. Sask), and within each DU, among the 24 HUC 8 watersheds. We also calculated a Euclidean distance matrix derived from the mean allele frequencies of the 24 HUC 8 watersheds; the relative strength of each proposed relationship was measured using bootstrap support values with the package *pvclust* (Suzuki and Shimodaira 2006) in R (R Development Team 2024) using 1000 bootstrap replicates as AU (Approximately Unbiased) *p*‐values (Figure S3). A dendrogram based on these distances was then plotted using *SNPRelate* (Zheng et al. 2012) in R (R Development Team 2024) to estimate the phylogenetic distance between bull trout in each watershed.

Isolation by distance (IBD) was assessed separately within each DU due to the disproportionate influence of large *F*_ST_ values between populations across the divide between the two DUs. Within each DU we then calculated IBD at two scales: specifically, we calculated pairwise geographic distance (in kilometers) between the midpoint coordinates (UTM N and E) of each HUC 8 watershed, and we used linearized *F*_ST_/(1 − *F*_ST_) values (Rousset 1997) to investigate IBD at the finest possible scale in our dataset. Secondly, we assessed pairwise waterway distances (in river kilometers) between the five HUC 2 watersheds using river segments in hydrology geoshapefiles within QGIS (Table 2). These geographic distances, along with pairwise linearized *F*_ST_/(1 − *F*_ST_) values between HUC 2 watersheds, were used in a separate broader‐scale IBD analysis to estimate how waterway distance correlates with genetic dissimilarity across the province. We used Mantel tests in the R package *vegan* (Oksanen et al. 2025) to evaluate IBD based on 999 permutations, and used the Bray‐Curtis method for estimating the genetic matrices and the Euclidean method for geographic matrices (Bray and Curtis 1957).

**TABLE 2:** Pairwise comparisons of *F*_ST_ (below diagonal line) for bull trout in five Hydrologic Unit Code (HUC) 2 watersheds, and waterway distance in km (above diagonal line) between each Hydrologic Unit Code (HUC) 2 river basin in Alberta Values in bold type indicate comparisons between watersheds across the divide between continental‐scale basins. Waterway distances are estimates using river segments from geoshapefiles in QGIS.

HUC 2 (River Basin)	Peace River	Athabasca River	North Sask. River	Red Deer River	South Sask. River
**Peace River**	0	2502.5	**10721.6**	**10698.8**	**10658.8**
**Athabasca River**	0.228	0	**11026.9**	**11004.1**	**10964.1**
**North Sask. River**	**0.450**	**0.506**	0	2863.6	2823.8
**Red Deer River**	**0.478**	**0.532**	0.213	0	1516.6
**South Sask River**	**0.513**	**0.590**	0.376	0.355	0

## 3 Results

After LD filtering, a total of 582 variable loci were used in the ADMIXTURE analysis of 962 BLTR samples across 24 HUC 8 watersheds in Alberta. The value of *K* that provides the most reliable ancestry estimates should be identifiable by an inflection point at the lowest cross‐validation (CV) error (Alexander et al. 2009). In our analysis, CV error declined continuously with increasing *K* without any substantial inflection point (Figure S2). This pattern suggests a hierarchical, or nested, population structure. Since our *ad hoc* ADMIXTURE‐based population identification did not reveal a clear “best” value of *K* corresponding to the most likely number of populations, we used a priori expectations of population differentiation between DUs (*K* = 2), among HUC 2 watersheds (*K* = 5), and among the finest‐scale watersheds in our dataset (*K* = 24 for HUC 8) for subsequent analyses and investigation of population boundaries.

**FIGURE 2:**
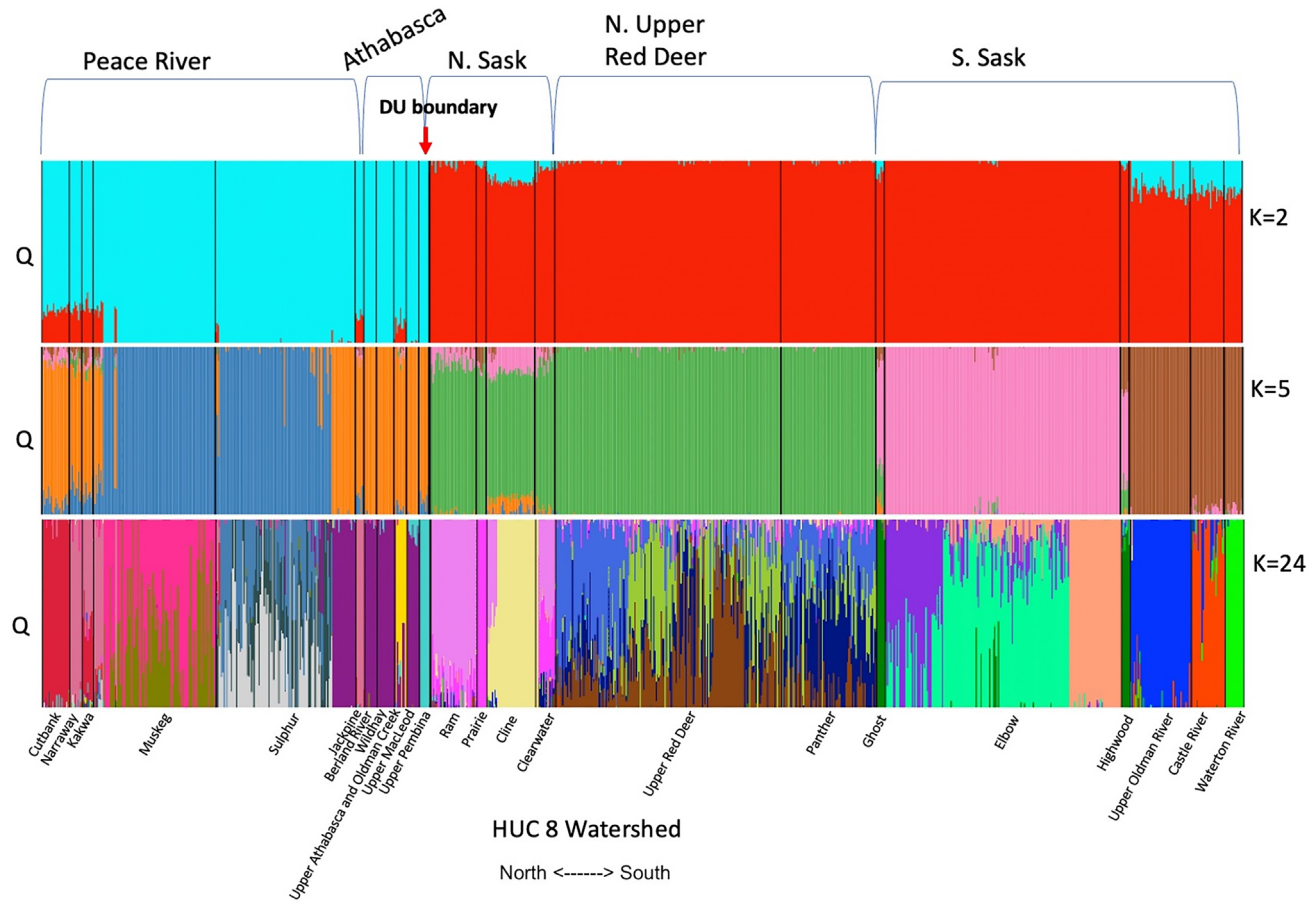
ADMIXTURE analysis of 962 bull trout genotypes across 24 Hydrologic Unit Code (HUC) 8 watersheds using *K* = 2 (based on DUs), *K* = 5 (based on HUC 2 watersheds), and *K* = 24 (based on HUC 8 watersheds). Watersheds are arranged left‐to‐right from north to south across Alberta.

**FIGURE 3:**
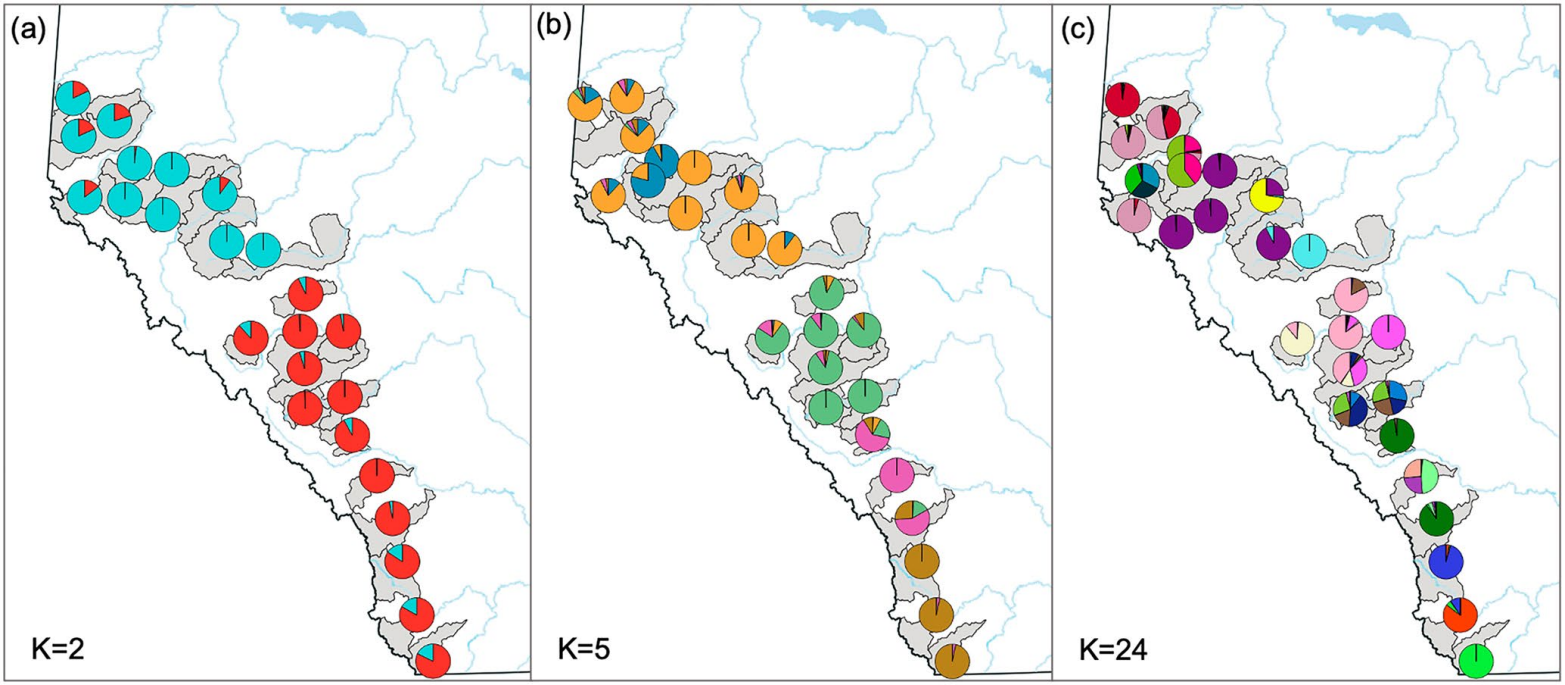
Average bull trout ancestry proportions (represented by colors) in 24 Hydrologic Unit Code (HUC) 8 watersheds using a priori population clusters (a) *K* = 2 (based on the two bull trout Designatable Units), (b) *K* = 5 (based on five distinct HUC 2 river basins), and (c) *K* = 24 (based on HUC 8 regional watersheds).

In every ADMIXTURE analysis (e.g., with *K* = 2, 5, or 24; Figures 2 and 3), there was clear evidence for discrete ancestry corresponding to the two continental‐scale basins in Alberta, and coinciding with the distribution of proposed DUs in the province: the Western Arctic DU and Saskatchewan‐Nelson DU (Figures 2 and 3a). There was not, however, a clear alignment between our a priori expectation of differentiation among the five HUC 2 watersheds and the ancestry estimates for *K* = 5 (Figures 2 and 3b). The two HUC 2 watersheds in the Western Arctic basin (i.e., the Peace River and Athabasca River watersheds) contain populations with a mix of ancestry mostly from two clusters (orange and blue in Figure 2 for *K* = 5), but those clusters had no clear geographic association (Figure 3). In the Saskatchewan‐Nelson basin, the North Saskatchewan HUC 2 watershed and the Red Deer River HUC 2 watershed contain populations that largely all share ancestry from a single cluster (green in Figure 2 for *K* = 5). The South Saskatchewan HUC 2 watershed contains populations with a mix of ancestry mostly from two clusters (pink and brown in Figure 2 for *K* = 5) corresponding to a North–South split with the watershed (Figure 3).

Setting *K* = 24 in our ADMIXTURE analyses revealed some distinct population clusters that coincide with HUC 8 watersheds, such as in the Waterton, Castle, Upper Oldman, Highwood, Ghost, Cline, Prairie, Ram, Upper Pembina, Upper Athabasca/Oldman Creek, Jackpine, and Cutbank (Figure 2). In other cases, however, HUC 8 watersheds contained mostly individuals with mixed ancestry, such as in the Muskeg, Sulfur, Clearwater, Upper Red Deer, and Panther River (Figure 2). There were also several cases of shared ancestry across HUC 8 watersheds, such as across the Cutbank‐Narraway‐Kakwa and across the Sulfur‐Berland‐Wildhay‐Upper Macleod (Figure 2). There was also evidence for a discrete population cluster at a smaller scale than the HUC 8 watershed in the Elbow (Figure 2). Overall, whereas our analysis reveals evidence for genetically distinct populations nested across many spatial scales, the clearest alignment of distinct clusters with a priori expectations was observed at the continental‐basin scale coinciding with the two DUs.

**FIGURE 4:**
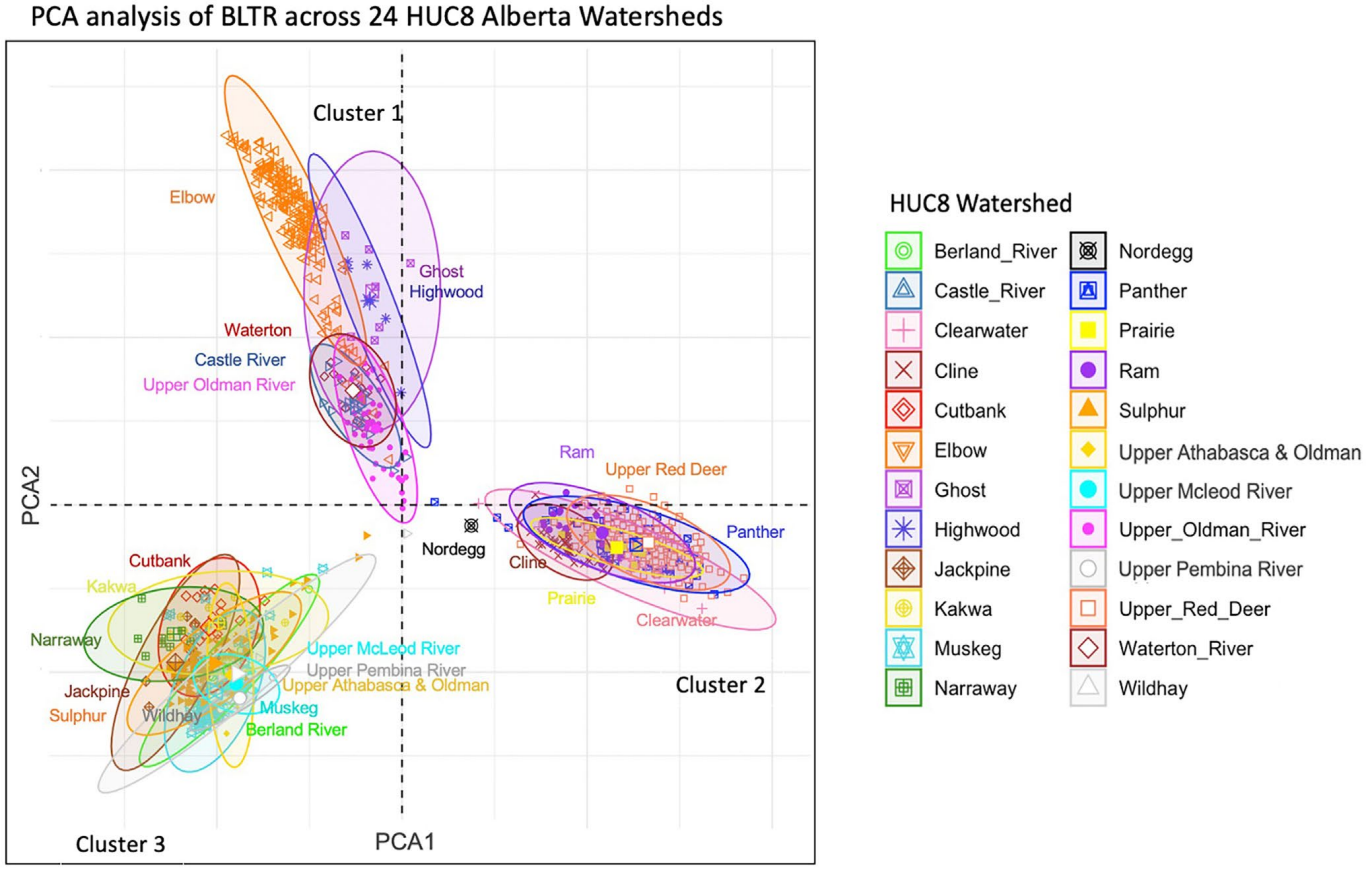
Principal component analysis (PCA) using genotypes of individual bull trout (*n* = 962) from 24 Hydrologic Unit Code (HUC) 8 watersheds along the Eastern Slopes of Alberta. The watersheds cluster as follows: Cluster 1 (top) corresponds to the South Saskatchewan River basin, Cluster 2 (right) to the Red Deer and North Saskatchewan River basins, and Cluster 3 (bottom left) to the Peace and Athabasca River basins.

**FIGURE 5:**
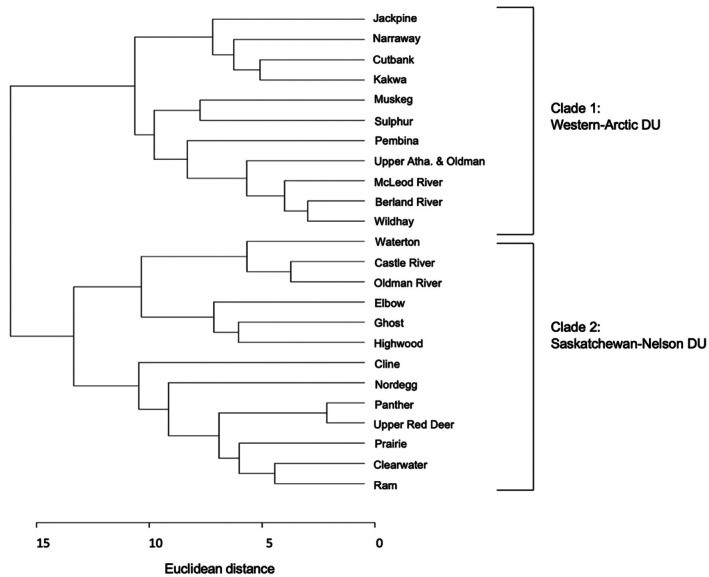
Dendrogram representation of a Euclidean distance matrix derived from mean allele frequencies of 24 Hydrologic Unit Code (HUC) 8 watersheds in Alberta. Clade 1 represents bull trout from the Western‐Arctic Designatable Unit, and Clade 2 represents bull trout from the Saskatchewan‐Nelson Designatable Unit.

Our PCA analysis revealed three distinct population clusters associated with major river basins: (i) the South Saskatchewan River basin, (ii) the Red Deer River and North Saskatchewan River basins, and (iii) the Peace River and Athabasca River basins (Figure 4). However, as PC1 explains ~12.57% of the total genetic variance and PC2 explains ~5.99%, only ~8.6% of the genetic variation observed is explained by the PCA. We did find a deep split between major river basins in the Saskatchewan‐Nelson DU in the dendrogram depicting Euclidean genetic distances between HUC 8 watersheds (Figure 5), which does correspond to the separation between Clusters 1 and 2 in our PCA plot (Figure 4). This indicates that two unique population clusters exist within the Nelson‐Saskatchewan DU, but that these two clusters do not correspond to the North and South Saskatchewan Rivers—instead grouping the bull trout found in the Red Deer River, which is a tributary of the South Saskatchewan River, unexpectedly with the bull trout in the North Saskatchewan River.

**FIGURE 6:**
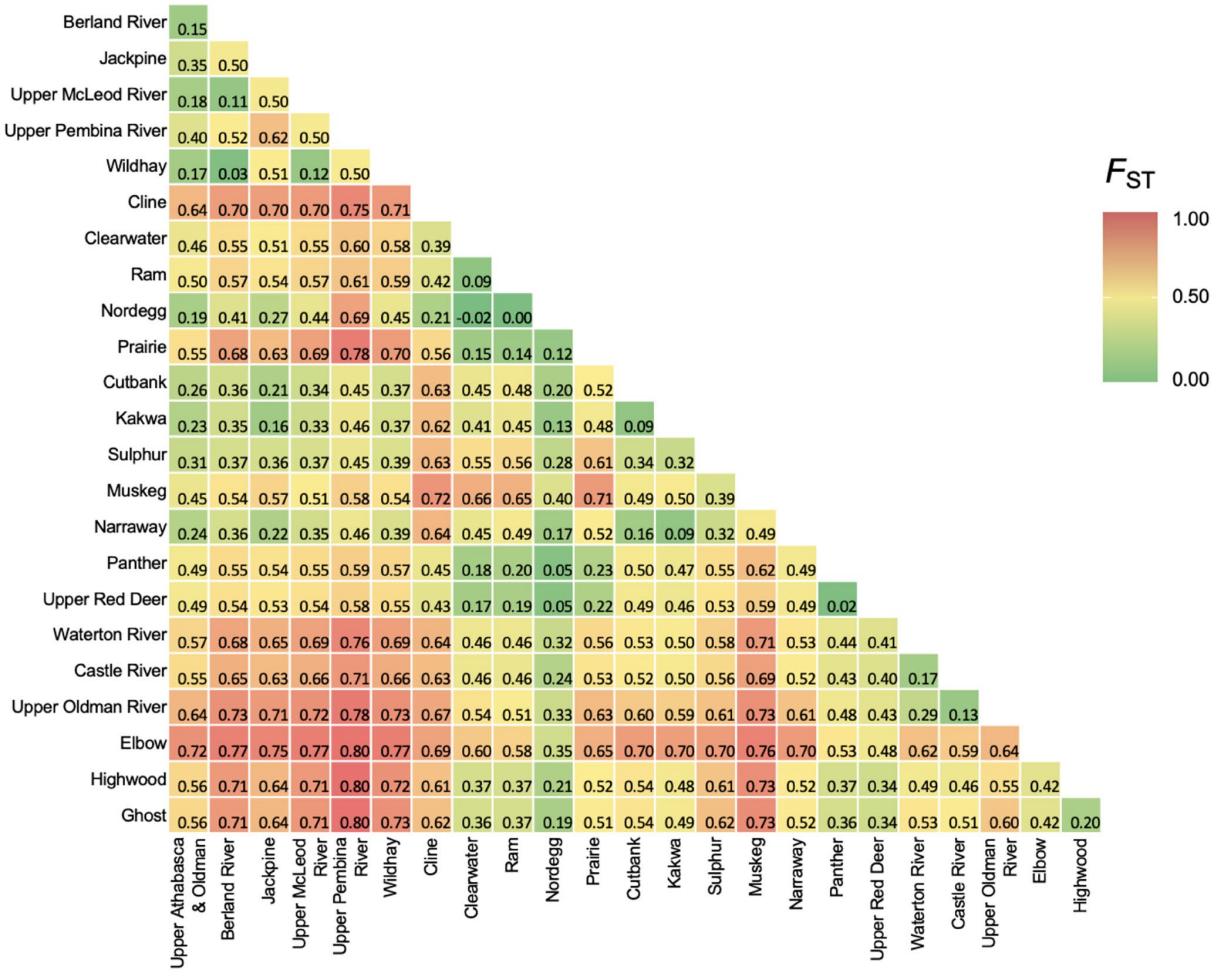
Heatmap of Pairwise *F*_ST_ between 24 Hydrologic Unit Code (HUC) 8 watersheds in Alberta.

**FIGURE 7:**
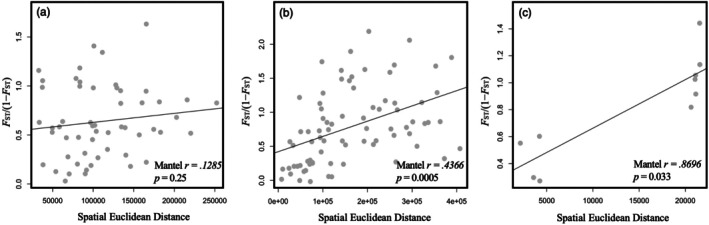
A pattern of isolation by distance was evaluated using a Mantel test at two scales: Across Hydrologic Unit Code (HUC) 8 watersheds within (a) the Western Arctic DU and (b) the Nelson‐Saskatchewan DU, and (c) across the five major HUC 2 river basins in Alberta.

The 95% confidence interval based on bootstrap replicates (*n* = 1000) for *F*_ST_ between samples from either side of the basin divide did not include zero, suggesting that the observed differentiation (*F*_ST_ = 0.400) between the Western Arctic and Saskatchewan‐Nelson basins is statistically significant (Figure S4). Pairwise *F*_ST_ values between the five HUC 2 watersheds (Table 2) and 24 HUC 8 watersheds (Figure 6) showed largest differentiation between northern‐most and southern‐most basins, suggesting differentiation is greatest between watersheds and basins that are most geographically distant from each other. In Figure 7, an increase in genetic distance was correlated with an increase in geographical distance within the Nelson‐Saskatchewan DU (*p* < 0.05, *r* = 0.4366; Figure 7b), but not within the Western Arctic DU (*p* = 0.25, *r* = 0.1285; Figure 7a). Genetic distance was significantly correlated with waterway distance at the HUC 2 scale across Alberta (*p* = 0.033, r = 0.8696; Figure 7c).

Expected heterozygosity (He), observed heterozygosity (Ho), and allelic richness (Ar) were assessed for each HUC 8 watershed (Table 1). Ho ranged from 0.0169 (Upper Pembina River) to 0.1149 (Narraway River), He ranged from 0.0168 (Upper Pembina River) to 0.1161 (Kakwa River), and allelic richness ranged from 1.03 (Upper Pembina River) to 1.32 (Muskeg River). The Upper Pembina River watershed had the lowest measures of diversity of all watersheds, whereas the Clearwater River, Kakwa River, and Narraway River exhibited the highest within population genetic diversity (i.e., He).

## 4 Discussion

To characterize the population structure and genetic diversity of bull trout across Alberta, we analyzed 582 SNPs in 962 individuals collected from the range of the bull trout distribution in the province. Our results reinforce the existence of strong genetic differentiation between bull trout in two continental‐scale basins, which corresponds to two previously identified DUs in Alberta: the Western Arctic DU and the Saskatchewan‐Nelson DU (COSEWIC 2020). Our *ad hoc* analysis using ADMIXTURE suggests that bull trout population structure below the scale of DUs is hierarchical, which corroborates previous findings on bull trout population structure in Alberta and elsewhere (Spruell et al. 2003; Warnock et al. 2010; Carroll and Vamosi 2021). There is substantial population genetic structure among watersheds at the sub‐basin scale, but there was inconsistent alignment between genetic population clusters and a priori expectations of population boundaries based on HUC 2 or HUC 8 watershed boundaries. Our analysis suggests, nonetheless, that some HUC 8 watersheds comprise genetically distinct and potentially locally adapted bull trout populations, such as in the Waterton River, Castle River, Upper Oldman River, Highwood River, Ghost River, Cline River, Prairie River, Ram River, Upper Pembina River, Upper Athabasca/Oldman Creek, Jackpine River, and Cutbank River. Bull trout recovery in Alberta would likely benefit from a conservation strategy similar to the bull trout recovery plan in the coterminous USA (USFWS 2015), which considers the hierarchical population structure of bull trout and promotes the maintenance of locally adapted populations.

Other distinct and potentially locally adapted populations likely exist at other geographic scales within and across HUC 8 watersheds, especially between populations of various life histories (fluvial, adfluvial, and resident), and across geographic barriers such as waterfalls. The distinct population segments identified in our *ad hoc* analysis are associated with landscape features that likely restrict gene flow and promote population differentiation. For example, in the Muskeg River and Sulfur River HUC 8 watersheds, distinct population structure is found between bull trout on either side of a main stem waterfall, suggesting potential restrictions to gene flow between fish above and below these barriers. Additionally, some distinct population clusters might be explained by past fish stocking or translocations, such as one example where bull trout were introduced to a fishless lake in the headwaters of the Sulfur River watershed from the adjacent Wildhay River watershed (Kissinger et al. 2024). A waterfall barrier below this lake restricts upstream movement of fish from the rest of the Sulfur basin (Kissinger et al. 2024), revealing a distinct population cluster with the same ancestry as the Wildhay in this upstream portion of the Sulfur River, despite being from a separate HUC 2 basin (purple in Figure 2 for *K* = 24). Fortunately, records of stocking and translocations of bull trout in Alberta (6 examples documented in Kissinger et al. 2024) and throughout North America (Hayes and Banish 2017) are rare relative to other salmonids (Halverson 2008) and few of these events resulted in established populations and thus have limited effects on the genetic structure of bull trout observed in our study. Lastly, a distinct bull trout population was found upstream in the headwaters of the Elbow River watershed, whereas the genetic distinctiveness seen here may be attributed to seasonal reductions in the flow of the main stem river (Manwell and Ryan 2006), resulting in isolation of bull trout populations in tributaries when water is low and connectivity via the main stem is limited.

As expected, bull trout exhibit significant population structure across their distribution at multiple scales (Spruell et al. 2003; Warnock et al. 2010) and fine‐scale population structure is common in bull trout (Al‐Chokhachy and Budy 2008; Warnock et al. 2010). One likely mechanism to finer‐scale population structuring is attributed to bull trout typically returning to their natal streams even after migrating long distances, thereby reducing gene flow (Barrows et al. 2016). Additionally, bull trout may have small breeding populations (Neraas and Spruell 2001; Whiteley et al. 2004), which contributes to population divergence via genetic drift (Leary et al. 1993; Kanda and Allendorf 2001; Whiteley et al. 2004; Barrows et al. 2016). Some connectivity between watersheds is likely maintained by occasional straying into non‐natal streams (Barrows et al. 2016), which is consistent with other salmonid species (e.g., Atlantic Salmon *Salmo salar* , Hagen et al. 2021 and Westslope cutthroat trout *Oncorhynchus clarkii lewisi* , Potvin et al. 2003; Taylor et al. 2003). The balance between these evolutionary processes (i.e., genetic drift and gene flow) is likely responsible for the significant pattern of isolation by distance in the Saskatchewan‐Nelson basin, as well as greater geophysical barriers, such as larger waterfalls and dams that contribute to restricted gene flow (Taylor and Warren Jr. 2001; Warnock et al. 2010). Conversely, in the Western Arctic basin, a lack of a significant pattern of isolation by distance was observed, especially in the Muskeg River and Sulfur River watersheds, which each appear to have multiple genetically discrete populations. A possible explanation for this increased genetic connectivity is likely a combination of 1. Increased elevation in the north portion of Alberta leading to greater thermal suitability for bull trout across these northern HUC 8 watersheds, and 2. a greater likelihood of bull trout expressing fluvial life histories, due to fewer dam constructions in these areas relative to southern Alberta. Since low dispersal and high levels of isolation likely drive the hierarchical population structure observed for bull trout (Ardren et al. 2011), conservation strategies should consider protecting these locally adapted populations while also protecting areas that promote dispersal to encourage the gene flow of bull trout.

Among all bull trout populations in our study, we observed the lowest genetic diversity in the Upper Pembina River, which is in the Athabasca River basin. This is consistent with the hypothesis that, similar to populations in Northeastern BC (i.e., in Pine Lake, Costello et al. 2003), the populations in the northern regions were among the last to be colonized following Pleistocene glacial retreat. There is evidence that there was a headwater connection during this time resulting in migratory pathways between the Fraser River and Athabasca River basins near Jasper National Park, Alberta (Nelson and Paetz 1992; Taylor et al. 2007; Tamkee et al. 2010), suggesting a possible migratory route for bull trout up the Fraser River into northern Alberta. In Southern Alberta, bull trout likely originated from populations in the Columbia River that migrated into southeastern portions of BC, and then into Alberta through postglacial lakes and rivers (Nelson and Paetz 1992). This may explain why bull trout in the Red Deer River were more closely related to those in the North Saskatchewan River than those in the South Saskatchewan River, despite the Red Deer River flowing into the South Saskatchewan River, and not the North. However, since postglacial lakes were widespread across Alberta towards the end of the Pleistocene (Utting 2019), multiple potential migration routes for bull trout into Alberta may have existed, leading to other migratory patterns that have not been accounted for in this study.

A source of bias in our study is the inconsistent sample size and methodology across each watershed sampled, thus, some inconsistency across each watershed limits our full understanding of bull trout migration across Alberta, especially regarding genetic diversity. Watersheds with samples pooled across multiple years or locations may be affected by the Wahlund effect, which may artificially lower observed diversity (Garnier‐Géré and Chikhi 2013) as a result of pooling multiple independent populations. Alternatively, the high genetic diversity observed in northern populations (i.e., Kakwa River and Narraway River watersheds) may reflect alternative historical dispersal pathways that are not explained from our study alone. Several other studies on the migratory pathways of other freshwater fishes into Alberta, such as the largescale sucker (*Catostomus macrocheilus)* and redside shiner ( *Richardsonius balteatus* ), indicate that colonization routes from the Columbia River extended into Alberta via proglacial lakes in and around the province (Nelson and Paetz 1992). For example, Westslope Cutthroat trout in southern Alberta are genetically similar to the “neoboreal” strain of westslope cutthroat trout found in Idaho and Montana, which originated in the upper Columbia Basin (Young et al. 2018). However, to fully understand these alternative evolutionary pathways, a more thorough phylogeographic assessment of bull trout across its entire range is needed.

Ultimately, in our study we contribute to the understanding that bull trout have two distinctive DUs as population distinctions are observed between the Athabasca and North Saskatchewan watersheds despite Alberta bull trout being derived by a single post‐glacier lineage (Carroll and Vamosi 2021). In our study, samples were collected opportunistically and inconsistently across watersheds, ranging from 1 sample in the Nordegg River watershed to 189 samples in the Elbow River watershed (Table 1). We note that while the single sample in the Nordegg River watershed is not representative of the watershed's overall population and should not be over‐interpreted as such, we found that removing this sample didn't change any general patterns, so this sample remained in our study to possibly understand which population(s) this sample is most similar to. Regardless, our inconsistent sampling methodology across all other watersheds may result in an imprecise estimate of genetic diversity from any watershed with small sample counts (< 10), and any interpretations within these populations are limited and provide an estimate of genetic diversity only.

Regardless, at the broadest scale, distinctive ancestral lines between the two bull trout DUs, the Western Arctic DU and Saskatchewan‐Nelson DU, are firmly evident (COSEWIC 2012). We also suggest that the 24 HUC 8 watersheds observed in this study have unique genetic variation and notable population structure within each finer‐scale watershed that are shaped by both the ecology of bull trout (i.e., affinity for natal homing), various bull trout life history stages (fluvial, adfluvial, and resident), impacts driven by geographic barriers, such as waterfalls and fragmentation of rivers (i.e., dams or culverts), which result in local population‐level structuring (Latham 2002), and historical evolutionary migration pathways. Consistently narrow population structure within watersheds suggests that bull trout across the province may be best described as a metapopulation with many unique local sub‐populations. As such, we suggest that protecting this organizational structure, such as through maintaining gene flow and preventing barriers to migration, may ultimately help conserve the unique bull trout genetic diversity that is essential for the persistence of the species (ASRD 2012).

## Author Contributions

**Emily R. Franks:** formal analysis (lead), investigation (lead), methodology (equal), writing – original draft (lead), writing – review and editing (equal). **Benjamin C. Kissinger:** conceptualization (lead), formal analysis (supporting), funding acquisition (equal), investigation (equal), methodology (equal), supervision (supporting), writing – review and editing (equal). **Steve Amish:** methodology (equal), resources (lead), validation (equal). **John R. Post:** conceptualization (supporting), funding acquisition (equal), investigation (supporting), project administration (lead), resources (equal), supervision (equal), writing – review and editing (supporting). **Jonathan A. Mee:** conceptualization (equal), formal analysis (supporting), methodology (equal), supervision (lead), validation (equal), writing – original draft (equal), writing – review and editing (equal).

## Funding

Funding was provided by the Office of the Chief Scientist, Government of Alberta.

## Conflicts of Interest

The authors declare no conflicts of interest.

## Supporting information


**Data S1:** ece372761‐sup‐0001‐supinfo.docx.

## Data Availability

All the required data are uploaded as Supporting Infromation only for review and not for publication.
