# Supplementary material for: Bull Trout (Salvelinus confluentus) Population Structure Across Alberta's Eastern Slopes
Source: Ecol Evol. 2026 Feb 24;16(2):e72761. doi: 10.1002/ece3.72761 (PMC12930102; doi:10.1002/ece3.72761)
Supplement: Supplementary file 1 — Data S1: ece372761‐sup‐0001‐supinfo.docx. [file ECE3-16-e72761-s001.docx]

**Bull Trout (*Salvelinus confluentus*) population structure across Alberta’s Eastern Slopes**

Emily R. Franks^1*^, Benjamin C. Kissinger^1,2^, Steve Amish^3^, John R. Post^1^, Jonathan A. Mee^1,4^.

^1^The University of Calgary; Calgary, AB, Canada.

^2^fRI Research; Hinton, AB, Canada.

^3^Montana Conservation Genomics Lab; Missoula, MT, USA.

^4^Mount Royal University; Calgary, AB, Canada.

^*^Corresponding author: Emily R. Franks (email: emily.franks@ucalgary.ca).

**Supplementary Material**


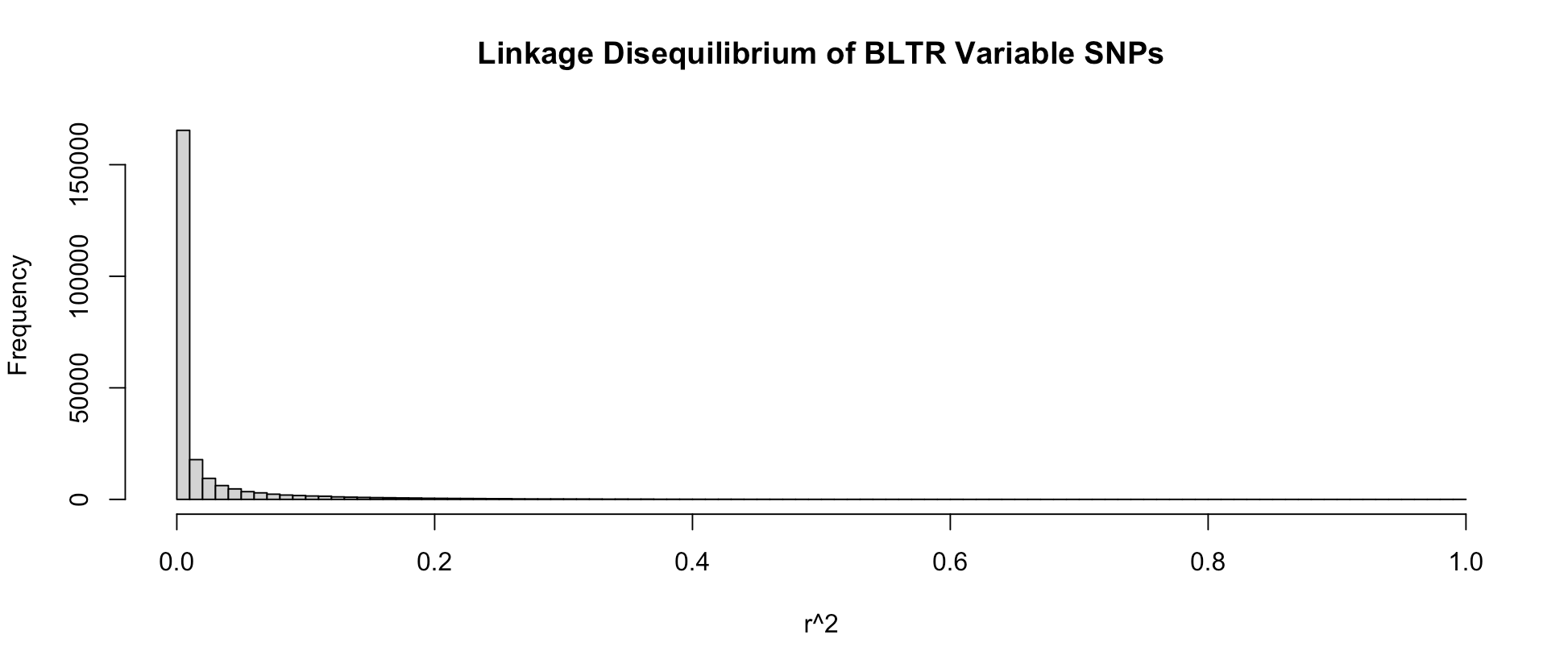


**Figure S1**. Pair-wise r^2^ values calculated from *genetics* package in RStudio for LD filtration on n = 1039 loci across the bull trout genome using n = 962 individual bull trout samples. Bull trout were collected across 24 Hydrologic Unit Code (HUC) 8 watersheds in Alberta between 1999-2022.

**Figure S2.** Cross Validation (CV) error scores of most probable K value for population assignment for SNP filtration from ADMIXTURE analysis (Alexander et al. 2009). Using genotypes of n = 962 bull trout collected from 24 Hydrologic Unit Code (HUC) 8 watersheds in Alberta, most probable K value is based on the lowest entropy score and inflection point, which was found at K=14 (0.08653); flat lining of additional K values suggests structure is most likely hierarchical beyond this value.


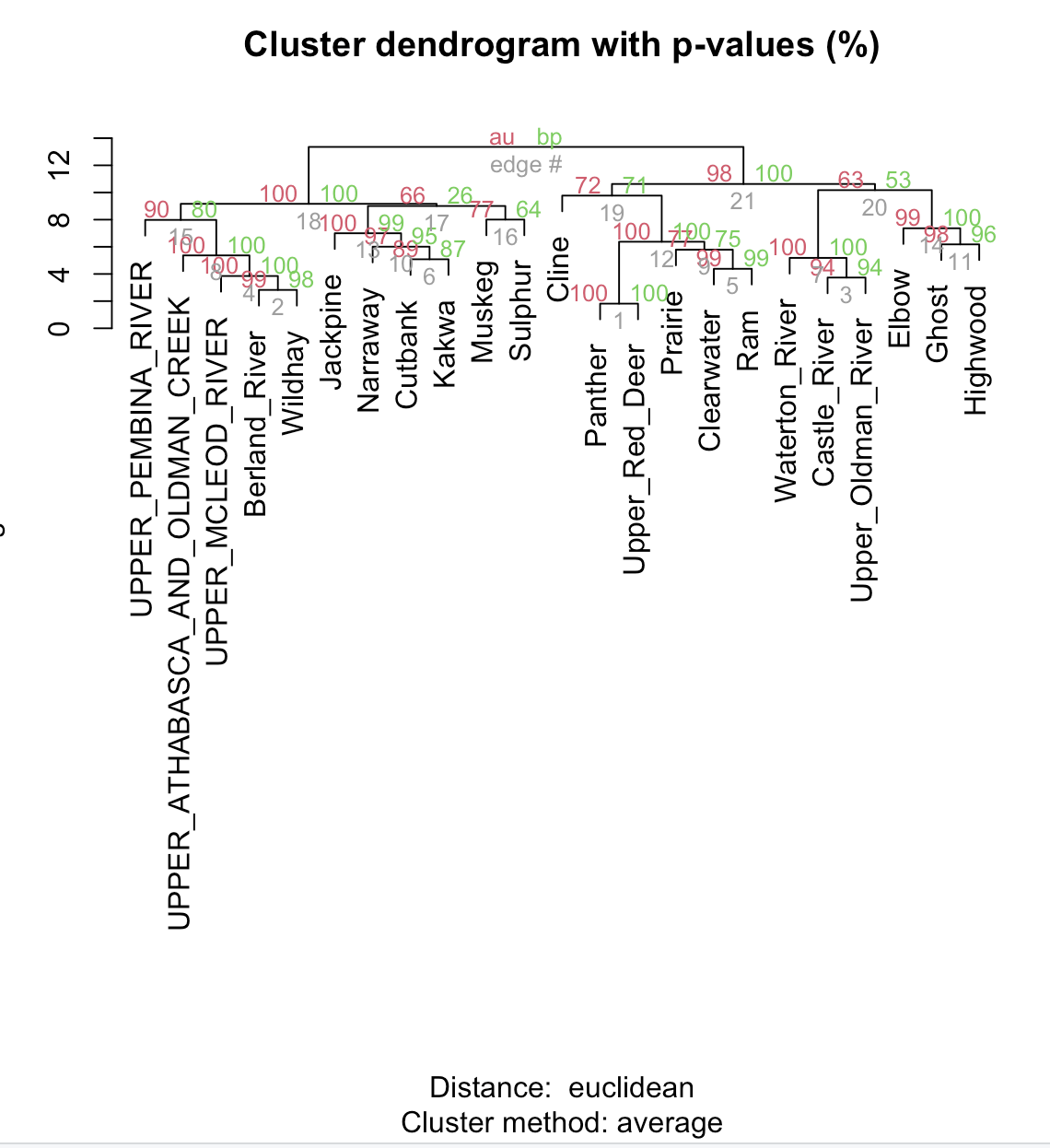


**Figure S3**: Cluster dendrogram of 24 Hydrologic Unit Code (HUC) 8 watersheds containing bull trout, in Alberta. The relative strength of each proposed relationship using bootstrap support values was measured with the ‘pvclust’ package using 1000 bootstrap replicates as AU (Approximately Unbiased) p-values.

**
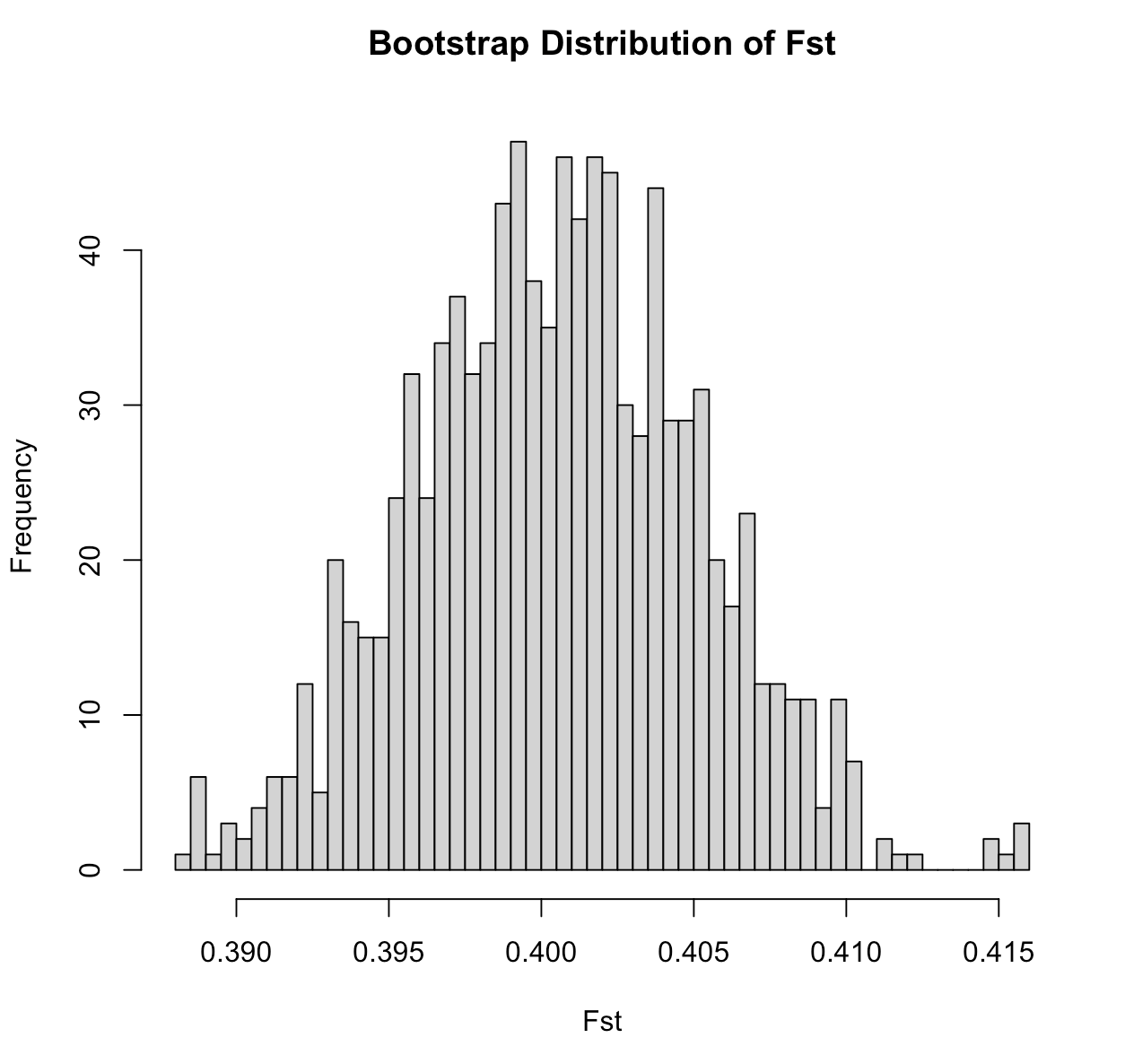
Figure S4.** Bootstrap (n = 1,000 replicates) of F_ST_ between Western Arctic DU and Saskatchewan-Nelson DU (average observed F_ST_ = 0.400) against the null hypothesis that F_ST_ = 0. F_ST_ was measured using the ‘Weir and Cockerham 1984’ method (Weir and Cockerham 1984) in the *adegenet* package (Jombart 2008) in R (R Development Team 2024).

**Table S1.** Dates and location numbers of BLTR samples collected in each HUC 8 watershed across the Eastern Slopes of Alberta. Years span 1998 – 2022.

| **HUC 8 Watershed** | **Dates samples collected:** | | **Number of locations:** |
| --- | --- | --- | --- |
|  | **From:** | **To:** |  |
| Berland River | 2017-07-10 | 2017-08-10 | 10 |
| Castle River | 2011-08-28 | 2012-10-17 | 27 |
| Clearwater | 2017-08-02 | 2020-10-17 | 16 |
| Cline | 2018-08-23 | 2019-09-18 | 39 |
| Cutbank | 2019-08-21 | 2019-08-22 | 22 |
| Elbow | 2017-08-10 | 2022-09-01 | 189 |
| Ghost | 2015-08-11 | 2016-08-09 | 7 |
| Highwood | 2017-08-17 |  | 7 |
| Jackpine | 2019-08-01 |  | 7 |
| Kakwa | 2017-07-07 | 2020-08-19 | 9 |
| Muskeg | 2022-07-09 | 2022-08-16 | 98 |
| Narraway | 2020-07-22 | 2020-08-06 | 10 |
| Nordegg | 1998-09-14 |  | 1 |
| Panther | 2017-06-29 | 2017-07-25 | 76 |
| Prairie | 2016-07-20 | 2016-07-26 | 8 |
| Ram | 2017-06-27 | 2018-07-12 | 37 |
| Sulphur | 2018-07-24 | 2022-07-31 | 112 |
| Upper Athabasca & Oldman Creek | 2014-08-11 | 2018-09-05 | 10 |
| Upper McLeod River | 2014-08-14 | 2018-08-29 | 10 |
| Upper Oldman River | 2017-08-02 | 2018-07-23 | 49 |
| Upper Pembina River | 2018-06-18 | 2020-07-31 | 8 |
| Upper Red Deer | 2017-07-13 | 2017-07-28 | 181 |
| Waterton River | 2019-07-30 | 2019-09-06 | 15 |
| Wildhay | 2012-08-03 | 2022-07-22 | 14 |
